# 

**Published:** 1859-04

**Authors:** 


VOL. II.] PUBLISHED MONTHLY. [No. 4.

### THE CHICAGO

### MEDICAL JOURNAL.

EDITED BY

DANIEL BRAINARD, M.D.,

Professor of Surgery in Rush Medical College; Surgeon to the U. S. Marine Hospital, etc.

[v.16]

**APRIL, 1859.**

CHICAGO:

JAMES BARNET, BOOK AND JOB PRINTER. 189 LAKE STREET.

AT TWO DOLLARS PER ANNUM, IN ADVANCE.

